# Targeted Plasma Metabolic Profiles and Risk of Recurrence in Stage II and III Colorectal Cancer Patients: Results from an International Cohort Consortium

**DOI:** 10.3390/metabo11030129

**Published:** 2021-02-24

**Authors:** Jennifer Ose, Biljana Gigic, Stefanie Brezina, Tengda Lin, Andreas Baierl, Anne J. M. R. Geijsen, Eline van Roekel, Nivonirina Robinot, Audrey Gicquiau, David Achaintre, Pekka Keski-Rahkonen, Fränzel J. B. van Duijnhoven, Tanja Gumpenberger, Andreana N. Holowatyj, Dieuwertje E. Kok, Annaleen Koole, Petra Schrotz-King, Alexis B. Ulrich, Martin Schneider, Arve Ulvik, Per-Magne Ueland, Matty P. Weijenberg, Nina Habermann, Augustin Scalbert, Andrea Gsur, Cornelia M. Ulrich

**Affiliations:** 1 Huntsman Cancer Institute Salt Lake City, Salt Lake City, UT 84112, USA; tengda.lin@hci.utah.edu (T.L.); Holowatyj@hci.utah.edu (A.N.H.); neli.ulrich@hci.utah.edu (C.M.U.); 2 Department of Population Health Sciences, University of Utah, Salt Lake City, UT 84112, USA; 3 Department of General, Visceral and Transplantation Surgery, University of Heidelberg, 1, 69117 Heidelberg, Germany; biljana.gigic@med.uni-heidelberg.de (B.G.); aulrich@lukasneuss.de (A.B.U.); martin.schneider@med.uni-heidelberg.de (M.S.); 4 Institute of Cancer Research, Department of Medicine I, Medical University of Vienna, 23, 1090 Wien, Austria; stefanie.brezina@meduniwien.ac.at (S.B.); tanja.gumpenberger@meduniwien.ac.at (T.G.); andrea.gsur@meduniwien.ac.at (A.G.); 5 Department of Statistics and Operations Research, University of Vienna, 1, 1010 Wien, Austria; andreas.baierl@univie.ac.at; 6 Division of Human Nutrition and Health, Wageningen University & Research, 6708 Wageningen, The Netherlands; annegeijsen@hotmail.com (A.J.M.R.G.); franzel.vanduijnhoven@wur.nl (F.J.B.v.D.); dieuwertje.kok@wur.nl (D.E.K.); 7 Department of Epidemiology, GROW-School of Oncology and Developmental Biology, Maastricht University, 30, 6229 Maastricht, The Netherlands; eline.vanroekel@maastrichtuniversity.nl (E.v.R.); annaleen.koole@maastrichtuniversity.nl (A.K.); mp.weijenberg@maastrichtuniversity.nl (M.P.W.); 8 Biomarkers Group, International Agency for Research on Cancer, 69372 Lyon, France; RobinotN@iarc.fr (N.R.); GicquiauA@iarc.fr (A.G.); achaintred@iarc.fr (D.A.); KeskiP@iarc.fr (P.K.-R.); ScalbertA@iarc.fr (A.S.); 9 Department of Medicine, Vanderbilt University Medical Center, Nashville, TN 37232, USA; 10 Vanderbilt-Ingram Cancer Center, Nashville, TN 37232, USA; 11 Division of Preventive Oncology, National Center for Tumor Diseases (NCT) and German Cancer Research Center (DKFZ), 460, 69120 Heidelberg, Germany; petra.schrotz-king@nct-heidelberg.de; 12 Klinik für Allgemein-, Viszeral-, Thorax- und Gefäßchirurgie, Städtische Kliniken Neuss, 84, 41464 Neuss, Germany; 13 BEVITAL, 87, 5021 Bergen, Norway; arve.ulvik@bevital.no (A.U.); per.ueland@ikb.uib.no (P.-M.U.); 14 Genome Biology, European Molecular Biology Laboratory (EMBL), 69117 Heidelberg, Germany; nina.habermann@embl.de

**Keywords:** colorectal cancer, recurrence, targeted metabolomics

## Abstract

The identification of patients at high-risk for colorectal cancer (CRC) recurrence remains an unmet clinical need. The aim of this study was to investigate associations of metabolites with risk of recurrence in stage II/III CRC patients. A targeted metabolomics assay (128 metabolites measured) was performed on pre-surgery collected EDTA plasma samples from *n* = 440 newly diagnosed stage II/III CRC patients. Patients have been recruited from four prospective cohort studies as part of an international consortium: Metabolomic profiles throughout the continuum of CRC (MetaboCCC). Cox proportional hazard models were computed to investigate associations of metabolites with recurrence, adjusted for age, sex, tumor stage, tumor site, body mass index, and cohort; false discovery rate (FDR) was used to account for multiple testing. Sixty-nine patients (15%) had a recurrence after a median follow-up time of 20 months. We identified 13 metabolites that were nominally associated with a reduced risk of recurrence. None of the associations were statistically significant after controlling for multiple testing. Pathway topology analyses did not reveal statistically significant associations between recurrence and alterations in metabolic pathways (e.g., sphingolipid metabolism *p* = 0.04; *pFDR* = 1.00). To conclude, we did not observe statistically significant associations between metabolites and CRC recurrence using a well-established metabolomics assay. The observed results require follow-up in larger studies.

## 1. Introduction

Colorectal cancer (CRC) remains a global public health issue being the third most prevalent cancer worldwide [1] and the fourth main global cause of cancer death with 881,000 deaths in 2018 [2]. About 142,000 individuals are estimated to be diagnosed with CRC and over 50,000 are estimated to die from CRC in the United States through 2020 [1]. Approximately 60% of CRC patients present with stage II/III disease [3]. Surgical resection is the primary course of treatment and only possible cure for these patients [4].

Although, the rate of recurrences in CRC patients has been reduced substantially over the past decades, there are still 20–30% of patients who are developing a postoperative recurrence, which results in poor survival outcomes [4,5,6] and is a major cause of morbidity and mortality [7]. The recurrence rate after curative surgery differs by tumor stage and is ~10% for patients diagnosed with stage II tumors and ~20% for patients diagnosed with stage III tumors [4]. To date, current treatment is largely based on clinical and pathologic parameters such as tumor stage, with little else to guide risk stratification of patients. As a result, issues of both overtreatment and undertreatment exist. At this time, the most accurate means for predicting prognosis for CRC remains the pathological stage, despite the fact that significant clinical heterogeneity in treatment response exists among patients with the same stage of cancer [8]. Clinical decision making will increasingly depend on the use of validated, high-quality biomarkers to guide surveillance and treatment of CRC patients and prevent unnecessary treatment. To date, clinically useful biomarkers predictive of recurrence for CRC patients are still lacking.

The metabolome reflects endogenous biological processes, environmental, and lifestyle factors [9,10,11,12]. Metabolomics assays can detect subtle differences in metabolism. Metabolomics is a powerful tool for analyzing metabolic alterations, and can provide sensitive and valuable diagnostic information, patient stratification and response to therapeutic treatment [13,14]. Metabolomics strategies may be targeted or un-targeted: targeted metabolomics (as used in the present study) defines the measurement of preselected, known metabolites, while untargeted metabolomics captures a broad range of small molecule compounds in a sample, including unknown compounds [13].

We conducted a multicenter, prospective study using biospecimens collected prior to surgery from *n* = 440 prospectively followed CRC patients from the international “Metabolomic profiles throughout the continuum of colorectal cancer” (MetaboCCC) consortium with an average follow-up time of 42 months to investigate associations of plasma metabolite concentrations with risk of recurrence in stage II/III CRC. We aimed to identify metabolites or a metabolic profile that are associated with recurrence and may improve postoperative prognostic stratification for patients diagnosed with stage II and stage III CRC.

## 2. Results

A total of *n* = 440 CRC patients were included in the present study (Table 1). Study endpoint is recurrence. Fifteen percent of patients were diagnosed with a recurrence (*n* = 69/440 patients). A total of 64% of patients with a recurrence (*n* = 44) presented with an early recurrence (occurred within 24 months after diagnosis). The average follow-up time of patients diagnosed with a recurrence was 19.9 months (range: 3.6–81.0 months).

Patients with recurrence were younger compared to patients without recurrence (63.0 years versus. 67.0 years; Table 2). Patients with a recurrence were more often diagnosed with stage III tumors (77% versus 54%) and did receive adjuvant treatment (54% versus to 40%) compared to patients without a recurrence. This is expected as adjuvant chemotherapy is standard of care for patients diagnosed with stage III tumors.

**Figure 1:**
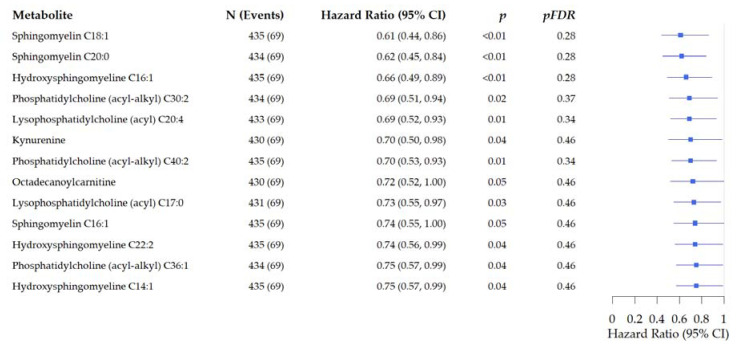
Forest plots of the associations of pre-surgery metabolite concentrations with risk of recurrence in prospectively followed colorectal cancer patients. Analysis is adjusted for age, sex, tumor stage, tumor site, BMI (kg/m^2^), and study cohort. (Hazard ratio and 95% Confidence Interval).

In overall analysis, thirteen metabolites including sphingomyelines, glycerophospholipds, lysophosphatidylcholines, and kynurenine were nominally associated with reduced risk of recurrence in CRC patients (Figure 1). Pathway topology analyses (PTA) did not show statistically significant associations between recurrence and alterations in sphingolipid metabolism (*p* = 0.04; *pFDR* = 1.00).

**Figure 2:**
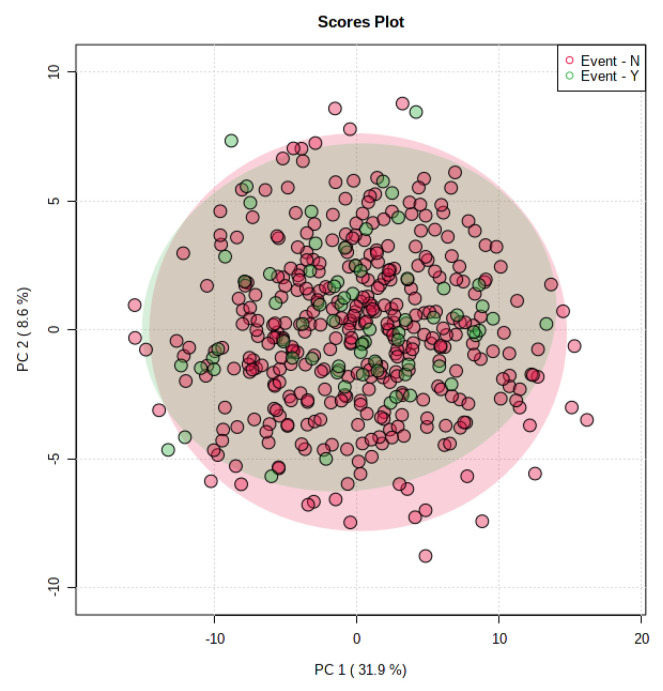
Principal component analysis plots derived from recurrent (green) and non-recurrent (red) colorectal cancer patients.

We performed principal component analysis using the metabolites identified in Figure 1. Those metabolites were not able to successfully discriminate between recurrent- and non-recurrent CRC patients. (Figure 2).

**Figure 3:**
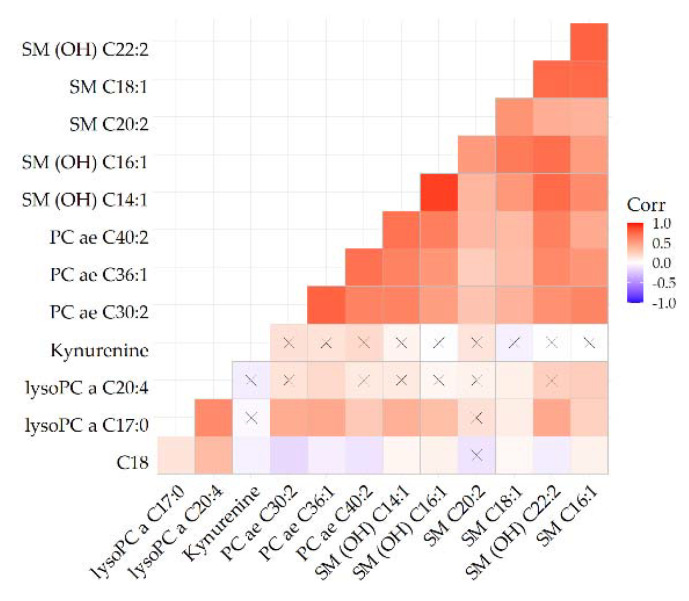
Heatmap depicting correlations of metabolites identified in Figure 1. For reference, blue indicates an inverse and red indicates a direct association. The stronger the color the stronger the correlation. Crossed cells indicate a non-significant correlation.

In a metabolite correlation heatmap, we identified one metabolite cluster including 8 of the 13 metabolites nominally associated with recurrence (Figure 3). This cluster included three glycerophospholipids, two sphyingomyelins, and four hydroxysphingomyelines. The correlations between metabolites in that cluster ranged from 0.22 to 0.89.

In analysis stratified by tumor location we observed inverse associations for 14 metabolites with risk of colon cancer recurrence. For example, hydroxysphingomyeline C16:1was associated with a 54% reduction of risk for colon cancer recurrence (HR: 0.46, 95% CI: 0.29–0.73, *p* < 0.01, *pFDR* = 0.06). PTA suggested three pathways to be related with risk of recurrence in colon cancer, although not significant after FDR adjustment: glycerophospholipid metabolism (*p* < 0.01, *pFDR* = 0.26), alpha-linoleic acid metabolism (*p* = 0.03, *pFDR* = 0.92), and linoleic acid metabolism (*p* = 0.01, *pFDR* = 0.54).

**Figure 4:**
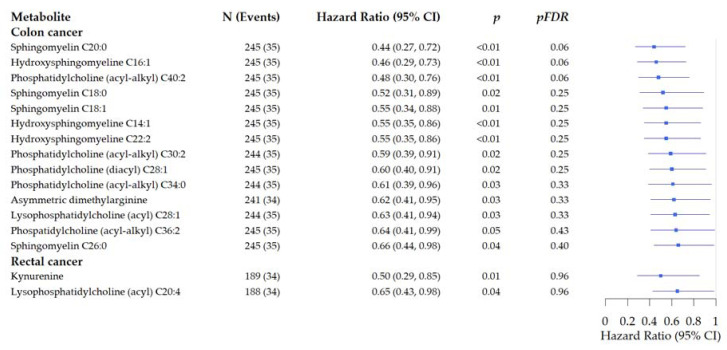
Forest plots of the associations of pre-surgery metabolite concentrations with risk of recurrence in prospectively followed colorectal cancer patients stratified by tumor site. Analysis are adjusted for age, sex, tumor stage, BMI (kg/m^2^), and study cohort. (Hazard ratio and 95% Confidence Interval).

Two metabolites were associated with reduced risk of rectal cancer recurrence (Figure 4). Kynureine was inversely associated with rectal cancer recurrence (HR: 0.50, 95% CI: 0.29–0.85, *p* = 0.01, *pFDR* = 0.96). PTA suggested glycerophospholipid metabolism to be related with risk of recurrence in rectal cancer, although not significant after FDR adjustment: glycerophospholipid metabolism (*p* = 0.04, *pFDR* = 1.00).

**Figure 5:**
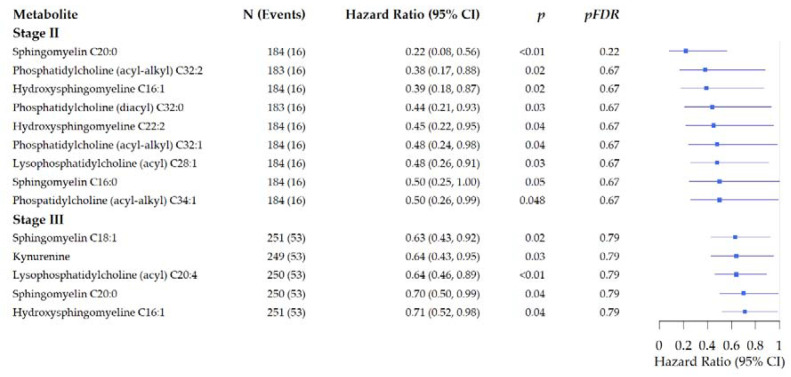
Forest plots of the associations of pre-surgery metabolite concentrations with risk of recurrence stratified by tumor stage in prospectively followed colorectal cancer patients. Analysis are adjusted for age, sex, tumor site, BMI (kg/m^2^), and study cohort. (Hazard ratio and 95% Confidence Interval).

In stratified analysis by tumor stage (Figure 5) we observed inverse associations of nine metabolites (including four glycerophospholipids and four sphingomyelins (*p* < 0.048, *pFDR* > 0.22) with risk of recurrence for patients diagnosed with stage II tumors. Glycerophospholipid pathway and sphingolipid metabolism were linked to stage II recurrence, but not after FDR adjustment (e.g., glycerophospholipid metabolism: *p* = 0.03, *pFDR* = 1.00). For patients diagnosed with stage III tumors we observed inverse associations for five metabolites including kynureine, and three sphingomyelines (*p* < 0.04, *pFDR* = 0.79). Two pathways that had been linked to recurrence in patients diagnosed with colon cancer were related to risk of recurrence in patients diagnosed with stage III tumors: linoleic acid metabolism (*p* = 0.02, *pFDR* = 0.67), alpha-linoleic acid metabolism (*p* = 0.04, *pFDR* = 1.00), and glycerophospholipid pathway (*p* < 0.01, *pFDR* = 0.42).

**Figure 6:**
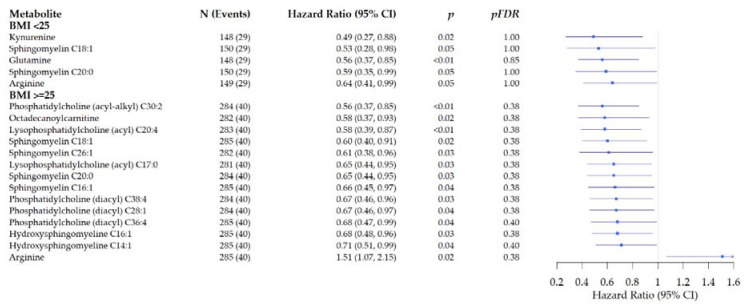
Forest plots of the associations of pre-surgery metabolite concentrations with risk of recurrence in prospectively followed colorectal cancer patients stratified by BMI at diagnosis. Analysis are adjusted for age, sex, tumor stage, tumor site, BMI (kg/m^2^), and study cohort. (Hazard ratio and 95% Confidence Interval).

In stratified analysis by BMI at diagnosis (Figure 6), we identified inverse associations for five metabolites with reduced risk of recurrence in patients with BMI <25, e.g., arginine: HR: 0.64, 95% CI: 0.41–0.99 (*p* = 0.05, *pFDR* = 1.00). Two pathways, D-glutamine and D-glutamate metabolism (*p* = 0.01, *pFDR* = 0.97) and arginine biosynthesis (*p* = 0.03, *pFDR* = 1.00) were marginally related to recurrence for patients with BMI < 25.

In patients with a BMI ≥ 25 at diagnosis, 14 metabolites were marginally associated with risk of recurrence (*p* < 0.04, *pFDR* = 0.38), of those one was associated with a slight increase in risk: arginine (e.g., arginine: HR: 1.51, 95% CI: 1.07–2.15 (*p* = 0.02, *pFDR* = 0.38). Four pathways were related to risk of recurrence in overweight and obese patients: glycerophospholipid metabolism (*p* < 0.01, *pFDR* = 0.53), linoleic acid metabolism (*p* = 0.01, *pFDR* = 0.54), alpha-linolenic acid metabolism (*p* = 0.04, *pFDR* = 0.75), and arginine biosynthesis (*p* = 0.04, *pFDR* = 0.75). Notable, an inverse association of arginine with risk of recurrence was observed in patients with a BMI < 25 at diagnosis, while an increase in risk of recurrence was observed in patients with a BMI ≥ 25 (Figure 6).

In sensitivity analysis limited to patients diagnosed with an early recurrence, we identified four metabolites nominally associated with risk of early recurrence including sphingomyeline C18:1 (HR: 0.56, 95% CI: 0.36–0.85, *p* < 0.01, *pFDR* = 0.76; Table 3). PTA suggested sphingolipid metabolism to be related with risk of recurrence in rectal cancer, although not significant after FDR adjustment (*p* = 0.03, *pFDR* = 1.00).

In additional sensitivity analysis, we excluded *n* = 17 samples from fasting patients. We observed robust results after excluding those samples from overall and stratified analysis (e.g., overall analysis: kynurenine: including fasting samples: HR: 0.70, 95% CI: 0.50–0.98, *p* = 0.04, *pFDR* = 0.48; excluding fasting samples: HR: 0.70, 95% CI: 0.50–1.00, *p* = 0.04, *pFDR* = 0.61; data not shown.)

As a final step, we did compare plasma metabolite concentrations between right- and left-sided colon cancers among female participants and did observe significant differences for 33 metabolites, predominantly glycerophospholipids, sphingomyelines, histidine, and tryptophan. We observed higher concentrations of all 33 identified metabolites in left-sided colon cancers compared to right-sided colon cancers (data not shown).

## 3. Discussion

To the best of our knowledge this is the first study investigating associations of pre-surgery plasma metabolite concentrations with risk of recurrence in prospectively followed CRC patients. We identified 13 metabolites (e.g., glycerophospholipids, sphingomyelins, and kynurenine) that were nominally associated with a reduced risk of colorectal cancer recurrence. In stratified analyses by BMI the metabolite arginine was nominally associated with increased risk of recurrence in patients overweight at the time of diagnosis. None of these associations were statistically significant after controlling for multiple testing.

To date, there are no other studies that have investigated associations of metabolites with risk of recurrence in CRC patients.

Using data from the same cohorts as presented here, we have previously characterized untargeted metabolic profiles from plasma samples using a discovery-replication strategy in colorectal cancer patients versus cancer-free controls [11]. Taurine, hypoxanthine, and two lyso-phosphatidylcholines were positively associated with colorectal cancer. In a subsequent publication we addressed differences in targeted metabolic profiles across different cancer stages and we found metabolites such as sphingomyelins, lysophosphatidylcholines, citrulline, and histidine to be associated with tumor stage [15].

The observed inverse, although not statistically significant, association of phopholipids and sphingomyelins with recurrence independent of tumor stage, tumor site, and BMI in the present study is notable.

Phosphatidylcholines and phosphatidylethanolamines [16] are key components of cell lipid bilayers [16], with direct impact on membrane structure and signaling pathways. It is plausible that changes in the composition of plasma glycerophospholipids may lead to improper signaling, and proliferation, as shown by in vitro and in vivo studies [17,18]. These mechanistic and pre-clinical data are supported by studies reporting positive associations of those metabolites with development of metastasis, and survival [17,19]. However, we did not identify other studies in cancer survivors using a metabolomics approach to investigate associations of metabolic profiles with recurrence in cancer patients. The observed inverse relationship in the present study needs further investigation in larger, prospective studies.

This study has strengths and limitations. This is the only prospective study to date investigating, among prospectively followed CRC patients, associations of metabolic profiles with risk of recurrence. Prior studies have been predominantly retrospective in nature and are less accurate in terms of data collections for the assessment of covariates for statistical modelling or have studied only few selected metabolites. Another challenge of the retrospective design is the inability to address reverse causation when it comes to clinically undiagnosed recurrence. While most colorectal cancer recurrences reportedly occur within 3 years following curative treatment, many studies are limited by short-term follow-up. In the present study we have long follow-up time for patients with a recurrence ranging between 3.6 months and 81.0 months. Out of the 69 patients with a recurrence, a total of *n* = 44 patients had a recurrence within 24 months after diagnosis. The present analyses are constrained to some degree by the modeling assumptions of the Cox proportional hazards model. While we expect the Cox models to be well-powered for detecting monotone (increasing or decreasing) relationships between metabolites and recurrence risk, the underlying relationships between metabolites and recurrence risk are complex, and likely contain non-linearity (on the log-hazard scale) and effect modifiers to some degree. For the present study we used a well-established and highly reproducible targeted metabolomics assay to measure metabolic profiles [20,21]. One disadvantage of utilizing a targeted approach is limited coverage of the metabolome, thus increasing the potential of missing metabolites of interest. The stability of metabolite concentrations using the Biocrates AbsoluteIDQTM p180 in serum and plasma samples processed within 24 h after blood collection and stored on cool packs has been demonstrated previously. [22] The samples from the present study were predominantly non-fasting (96%), though prior data suggest that few metabolites are strongly related (>10% of variance explained) to recent food intake. [23] The sample size for the present study was sufficiently powered for overall analyses, however, limited for subgroup analyses by tumor stage, tumor site, and BMI which are exploratory in nature.

In summary, we did not observe statistically significant associations between metabolites included in the Biocrates *AbsoluteIDQ*^TM^ p180 kit and CRC recurrence. However, nominally significant metabolites identified in the present study require follow-up in larger settings with more statistical power for subgroup analysis by tumor stage, tumor site, or BMI.

## 4. Materials and Methods

Targeted metabolomics assays (Biocrates *AbsoluteIDQ*^TM^ p180Kit, Innsbruck, Austria) were performed on pre-surgery collected plasma samples from *n* = 440 newly diagnosed stage II/III colorectal cancer patients recruited within four prospective cohort studies from the international consortium “Metabolomic profiles throughout the continuum of colorectal cancer” (MetaboCCC) [11,15]. Patients with a recurrence of primary colorectal cancer at least three months after curative surgery were defined as event.

### 4.1. Study Design and Populations

Data from four cohort studies embedded in the MetaboCCC consortium, a large consortium of European CRC survivor cohorts to investigate metabolic profiles across the continuum of colorectal cancer carcinogenesis, were included. The four cohorts are: (1) the COLON study [24] from the Netherlands (ClinicalTrials.gov Identifier: NCT03191110), (2), the EnCoRe study [25] from the Netherlands (Netherlands Trial Register: 7099), (3) the Heidelberg site of the international ColoCare Study [26] (ClinicalTrials.gov Identifier: NCT02328677) and (4) the Colorectal Cancer Study of Austria (CORSA). All cohorts were approved by local Medical Ethics Committees and all participants provided written informed consent. In total, *n* = 440 CRC patients were included in the current study of which *n* = 135, *n* = 137, *n* = 142 and *n* = 26 of the COLON, EnCoRe, ColoCare, and CORSA cohort, respectively. The COLON study is a prospective cohort study in the Netherlands which started in 2010 [24]. Participants were recruited at diagnosis from eleven hospitals in the Netherlands. The EnCoRe study, initiated in 2012, is an ongoing prospective cohort study [25]. CRC patients were recruited at diagnosis from three hospitals in the south-east of the Netherlands. The ColoCare Study is an ongoing, international, multi-centre prospective study which started in 2007, the ColoCare Study site in Germany started recruitment in 2010 [26]. In this analysis we used data from patients recruited at the University Hospital of Heidelberg were included. CORSA is an ongoing study recruiting colorectal cancer patients in cooperation with the province-wide screening project Burgenland Prevention Trial of Colorectal Disease with “Immunological Testing” (B-PREDICT), since 2003, using fecal immunochemical test (FIT). FIT-positive tested individuals subsequently received a complete colonoscopy. Additional colorectal cancer patients were recruited at four hospitals in Vienna.

The inclusion and exclusion criteria of the individual cohorts have been published previously [24,25,26]. All participants had histologically confirmed colorectal cancer and had data on targeted metabolomics data from pre-surgery collected plasma samples available. Patients were eligible for the present study if they were diagnosed with stage II/III colorectal cancer and had surgery.

### 4.2. Data Collection

In all cohorts, EDTA plasma samples were collected upon recruitment, i.e., shortly after colorectal cancer diagnosis, at the time of colonoscopy, and mostly before surgical or chemo and/or radiation treatment for colorectal cancer. Generally, plasma samples were collected and processed within four hours after withdrawal and stored at the corresponding study sites at −80 °C.

Clinical data, including TNM-stage, tumor site, and treatment characteristics, e.g., surgery date, neo-adjuvant and adjuvant chemo- and-/or radiation therapy, were abstracted from medical records for all cohorts. Both the pathological and clinical TNM characteristics, i.e., pathological Tumor-Node-Metastasis (pTNM) and clinical Tumor-Node-Metastasis (cTNM), were collected from medical records; pTNM was used for staging for patients with colon cancer receiving surgery while cTNM was used for all rectal cancer patients with neo-adjuvant therapy. Patients were staged according to the TNM classification of Malignant Tumors of the Union for International Cancer Control (8th version, 2016) [27].

Demographic and lifestyle characteristics, including age at diagnosis, sex, body mass index at diagnosis (BMI), were collected through study-specific questionnaires. All clinical, demographic and lifestyle data were harmonized across the cohorts included in the MetaboCCC consortium. The study end point investigated here was recurrence. Recurrence was defined as locoregional or distant recurrence after complete tumor resection. Early recurrence is defined as recurrence that occurred within 24 months after diagnosis, late recurrence is defined as recurrence that occurred 24 months after diagnosis.

### 4.3. Biomarker Analysis

Non-fasting (*n* = 423) and fasting blood samples (*n* = 17) were selected from all cohorts. All samples were shipped on dry ice and analyzed at the International Agency for Research on Cancer (IARC) in Lyon, France using the targeted metabolomics *AbsoluteIDQ*^TM^ p180 kit (BIOCRATES Life Sciences AG, Innsbruck, Austria). EDTA plasma samples had zero or one freeze-thaw cycle at time of measurement; COLON, EnCoRe and CORSA plasma samples were thawed once while ColoCare samples were not thawed before shipment to IARC. All samples of each cohort were analyzed subsequently over a total of 19 batches between May and October 2016.

The kit (semi-)quantifies up to 188 metabolites from five compound classes: 21 amino acids, 21 biogenic amines, 90 glycerophospholipids, 15 sphingomyelins, 40 acylcarnitines and sum of hexoses. The analytical procedure has previously been described in detail [20,21].

Ultra-performance liquid chromatography (UPLC) coupled to a tandem mass spectrometer (MS/MS) was used, following the manufacturer’s recommendations. Methanol (LC-MS Chromasolv, Honeywell SA, St Priest, France) was used for chromatography. All other chemicals and standards were provided with the kit by Biocrates. Amino acids and biogenic amines were (semi-) quantified by UPLC-MS/MS. Samples are analyzed with a UHPLC-MS/MS system consisting of a 1290 binary LC pump (Agilent, Santa Clara, CA, USA) and an API4500 triple quadrupole mass spectrometer (Sciex, Framingham, MA, USA). Autosampler tray is kept refrigerated at 10 °C. A system suitability test (SST) is conducted before injecting the samples with two test mix provided with each kit, one for LC-MS/MS analyses (mix of derivatized amino acids and biogenic amines) and one for FIA (mix of lipids, acylcarnitines and hexose). The test is intended to check MS instrument sensitivity, chromatographic conditions and retention times. Lipids, sugar and acylcarnitines were analysed by flow injection (FIA)-MS/MS [19]. Samples (5 µL) are injected on an Acquity UPLC BEH C18 column (2.1 × 75 mm, 1.7 µm; Waters, Milford, MA, USA) mounted with a guard column Acquity BEH C18 VanGuard (2.1 × 5 mm, 1.7 µm; Waters). Column temperature is 50 °C. Flow injection analysis does not allow separating isobaric compounds. Each measured species may thus be a mixture of several lipid or acylcarnitine isomers.

For the LC part, isotope-labelled internal standards are provided for all amino acids and biogenic amines for calibration. Seven concentration levels of standard mixes are provided as lyophilized material, each amino acid and biogenic amine are quantified against the calibration curves. Twenty-nine isotope-labelled internal standards are used for quantifying 42 metabolites. Regarding the FIA part, lipids are “semi-quantified” as no calibrations with standards are performed, and the quantification is based on only 10 isotope-labelled internal standards for the 41 acylcarnitines and hexose and on 4 non-istotope labelled internal standards for the other lipids (glycerophospholipids and sphingomyelins). For semi-quantified analytes, only lower limit of detection (LOD) is given as no calibration is made.

Each plate from the kit included three wells with phosphate buffer saline (PBS), used as a zero sample, seven wells with increasing concentration levels of standard mixes of amino acids and biogenic amines for calibration, as well as three quality control samples (QCs) supplied by Biocrates. All samples were analyzed once. Samples were randomized per cohort, and samples from each cohort were analyzed in sequential order on multiple plates. Internal standards, PBS samples, calibration samples for amino acids and biogenic amines as provided by Biocrates, plasma samples and a pooled internal QC sample (10 µL) are added to the wells. The plate is then dried during 30 min under vacuum. Phenylthiocarbamyl derivatives of amino acids and biogenic amines are formed by adding phenylisothiocyanate reagent. After 20 min the plate is dried for 2 h under vacuum. The analytes are extracted with a solution of 5 mM ammonium acetate in methanol (shook for 30 min at 450 rpm) and the extracts filtered by centrifugation (2 min at 500 g). Samples are diluted as follows before MS analysis: (1) for UPLC-MS/MS, dilution 1:5 with methanol-water 40:60 and (2) For FIA-MS/MS, dilution 1:20 with the “FIA elution solvent”. QCs were lyophilized human plasma samples, to which 59 metabolites had been spiked at three concentration levels. In addition, two IARC QC samples (QC1 and QC2) were analyzed in duplicate in each 96-well plate. These QCs were two citrate plasma samples.

Metabolites with inter- or -batch coefficients of variation (CVs) >20% using quality control samples were excluded from the present analysis. Subsequently, metabolites with >20% of missing values across all cohorts, including values below or above the LOD or limit of quantification (LOQ), whichever is applicable, were excluded.

After quality control, a total of 128 metabolites were retained for further analysis. These included 76 phospholipids, 19 amino acids, 7 biogenic amines, 14 sphingolipids, 11 acylcarnitines, and 1 hexoses (Supplementary Table S1). For those 128 metabolites, imputation was used to replace missing values (<20%) across all cohorts. Values below the LOD, were imputed by half of the batch-specific LOD, while values below the lower LOQ were imputed by the LOQ. Values above the upper LOD, were set at the upper limit. These procedures were chosen to be in line with what has been applied in previous studies using data of the same kit [21,28].

Chromatographic peaks (UPLC-MS/MS analyses) were integrated with the MultiQuant Software (AB Sciex, Framingham, MA, USA) and exported into the MetIDQ software (Biocrates Life Sciences AG, Innsbruck, Austria). For FIA-MS/MS analyses, files were directly exported to MetIDQ software to be parsed. The instrumentation consisted of a Sciex Triple Quad 4500 MS with an electrospray ion source and an Agilent Infinity 1290 UHPLC system with a Waters Acquity UPLC BEH C18 (1.7 µm, 2.1 × 75 mm) column and VanGuard (1.7 µm, 2.1 × 5 mm) precolumn.

### 4.4. Statistical Analysis

Patients’ demographical and clinical characteristics were described for patients with and without a colorectal cancer recurrence. Metabolites with missing values in >50% of either patients with an event or patients without an event were excluded from analysis. Metabolite intensities were log2 transformed prior to statistical analysis, to prevent heteroscedasticity [29]. Outliers were identified using the extreme studentized deviate (ESD) many-outlier approach and excluded from subsequent analysis [30]. Demographic and clinical characteristics are presented as medians and range, or as numbers with corresponding percentages. Body mass index (BMI) was calculated as weight (kg) divided by the square of height (m^2^) for all cohorts, with the exception of the ENCORE study that collected information on height and weight by trained dieticians. BMI status was categorized based on the recommendations from the World Health Organization (WHO): underweight (<18.5 kg/m^2^), normal weight (18.5–24.9 kg/m^2^), overweight (25.0–29.9 kg/m^2^) and obese (≥30.0 kg/m^2^).

Cox proportional hazard models were computed to investigate associations of metabolites with recurrence adjusted for age, sex, cohort, tumor stage, tumor site, body mass index, and cohort; false discovery rate (FDR) was used to account for multiple testing [31]. Log two standardized hazard ratios (HR) and 95% confidence intervals (CIs) were calculated using cox proportional hazard models with the clinical outcome (recurrence) as dependent variable to test the association with plasma metabolites. The HR represents the change in risk for colorectal cancer recurrence when a one standard deviation (SD) change in metabolite intensity, allowing comparison of effect sizes between different features. Analysis was adjusted for age at diagnosis, sex, tumor site, tumor stage, BMI (continuous), and cohort. A FDR *p*-value <0.05 was considered statistically significant. Subgroup analyses were conducted to assess potential effect modification by tumor location, tumor stage, and BMI at diagnosis (BMI < 25 and BMI ≥ 25). Cox proportional hazard models were computed in SAS 9.4 (Cary, NC, USA) using two-sided tests. A *p*-value of <0.05 was considered statistically significant. Pathway topology analyses (PTA) were performed to identify biologically relevant pathways if more than three metabolites were significantly associated with risk of recurrence, before adjustment for multiple testing (MetaboAnalyst 5.0) [32]. We used principal component analysis to assess potential clustering of samples and weather identified metabolites are able to discriminate between recurrent and non-recurrent cases. We further prepared a heatmap to visualize the correlations across metabolites that were nominally significant in overall analysis. We further prepared a heatmap to visualize the correlations across metabolites that were nominally significant in overall analysis.

## Figures and Tables

**Table 1:** Baseline characteristics of the overall study population and by cohort.

COHORTS					
Participant Characteristics-	Total Population	COLON	EnCoRe	CORSA	ColoCare
Number of participants	440	135	137	26	142
Sex, *n* (%)					
Male	282 (64%)	76 (56%)	86 (63%)	22 (84%)	98 (69%)
Female	158 (36%)	59 (44%)	51 (37%)	4 (16%)	44 (31%)
Age at diagnosis, years (median, range)	66.0 (27.0–88.0)	66.0 (31.0–84.0)	67.0 (36.0–87.0)	69.5 (39.0–88.0)	64.0 (27.0–86.0)
Body mass index *					
Continuous, kg/m^2^ (median, range)	26.3 (17.2–46.0)	25.4 (17.2–40.2)	27.2 (19.0–46.0)	26.4 (21.0–35.9)	26.2 (17.8–39.7)
Underweight, <18.5, *n* (%)	5 (1%)	3 (2%)			2 (1%)
Normal weight, 18.5–24.9, *n* (%)	143 (33%)	57 (44%)	36(26%)	6 (26%)	44 (31%)
Overweight, 25–29.9, *n* (%)	192 (45%)	52 (40%)	58 (43%)	13 (57%)	69 (49%)
Obese, ≥30, *n* (%)	92 (21%)	19 (14%)	42 (31%)	5 (17%)	26 (19%)
Tumor stage, *n* (%)					
II	187 (43%)	66 (49%)	45 (33%)	13 (50%)	63 (44%)
III	253 (57%)	69 (51%)	92 (67%)	13 (50%)	79 (56%)
Tumor location ^1^, *n* (%)					
Colon cancer	248 (57%)	86 (64%)	81 (59%)	17 (71%)	64 (45%)
Distal colon	129 (30%)	44 (33%)	39 (28%)	10 (42%)	36 (25%)
Proximal colon	119 (27%)	42 (31%)	42 (31%)	7 (29%)	28 (20%)
Rectal cancer	190 (43%)	49 (36%)	56 (41%)	7 (29%)	78 (55%)
Neo-adjuvant treatment, *n* (%)					
Yes	306 (70%)	91 (67%)	90 (66%)	25 (96%)	100 (70%)
No	134 (30%)	44 (33%)	47 (34%)	1 (4%)	42 (30%)
Adjuvant treatment, *n* (%)					
Yes	183 (42%)	42 (32%)	64 (47%)	12 (46%)	65 (47%)
No	249 (58%)	90 (68%)	73 (53%)	14 (54%)	72 (53%)
Follow-up time (months), median, range)					
Patients with recurrence	19.9 (3.6–81.0)	27.1 (7.0–81.0)	15.0 (3.7–45.1)	46.7 (39.0–65.8)	15.7 (3.6–45.6)
Patients without recurrence	42.2 (3.6–88.8)	71.2 (9.3–88.8)	41.9 (4.1–69.1)	44.9 (3.6–70.1)	31.5 (3.6–65.8)
Recurrence ^2^, *n* (%)					
Yes	69 (15%)	20 (15%)	23 (17%)	6 (23%)	20 (14%)
Early Recurrence ^3^	44 (64%)	8 (40%)	18 (78%)		18 (90%)
Late Recurrence ^4^	25 (36%)	12 (60%)	5 (22%)	6 (100%)	2 (10%)
No	371 (85%)	115 (85%)	114 (83%)	20 (77%)	122 (86%)

* Body Mass Index (BMI) in all cohorts was based on self-reported height and weight. Except, in the ENCORE cohort height and weight have been assessed by a trained dietician. ^1^ Tumor location is defined as colon (cecum, appendix and ascending colon, hepatic flexure, transverse colon, splenic flexure, descending colon and sigmoid colon) and rectal (rectosigmoid junction and rectum) cancer. ^2^ Recurrence is defined as colorectal cancer recurrence (event) in patients who had complete tumor resection. ^3^ Early recurrence is defined as recurrence <= 24 months after diagnosis. ^4^ Late recurrence is defined as recurrence that occurs >24 months after diagnosis.

**Table 2:** Baseline characteristics of patients with and without a recurrence.

Participant Characteristics-	Patients without Recurrence	Patients with Recurrence
Number of participants	371	69
Sex, *n* (%)		
Female	137 (37%)	21 (30%)
Male	234 (63%)	48 (70%)
Age at diagnosis, years (median, range)	67.0 (27–88)	63.0 (38–87)
Tumor stage, *n* (%)		
II	171 (46%)	16 (23%)
III	200 (54%)	53 (77%)
Tumor location, *n* (%)		
Colon	213 (58%)	45 (51%)
Distal colon	104 (28%)	15 (21%)
Proximal colon	109 (30%)	30 (29%)
Rectal	156 (42%)	34 (49%)
Neo-adjuvant treatment, *n* (%)		
Yes	110 (30%)	24 (35%)
No	261 (70%)	45 (65%)
Adjuvant treatment, *n* (%)		
Yes	147 (40%)	36 (54%)
No	218 (60%)	31 (46%)

**Table 3:** Associations of pre-surgery metabolite concentrations with risk of early recurrence (recurrence occurred within 24 months after diagnosis) in prospectively followed colorectal cancer patients. Analysis is adjusted for age, sex, tumor stage, tumor site, BMI, adjuvant treatment, and study cohort.

Metabolite	N Patients ^1^ (N Events ^2^)	HR (95% Confidence Interval)	*p*	*pFDR*
Lysophosphatidylcholine (acyl) C20:4	438 (44)	0.67 (0.47, 0.96)	0.03	0.76
Hydroxysphingomyeline C16:1	438 (44)	0.69 (0.48, 0.99)	<0.01	0.76
Sphingomyeline C18:1	438 (44)	0.56 (0.36, 0.85)	<0.01	0.76
Sphingomyeline C20:2	438 (44)	0.67 (0.46, 0.97)	0.03	0.76

^1^ Patients who were not diagnosed with a recurrence during the study follow-up. ^2^ Patients who were diagnosed with a recurrence during study follow-up.

## Data Availability

Study data are available from the PIs of the respective studies on reasonable request. Please contact the corresponding author for further information.

## Supplementary Materials

The following are available online at https://www.mdpi.com/2218-1989/11/3/129/s1, Table S1: Metabolite classes and names.
