# Supplementary material for: Targeted Plasma Metabolic Profiles and Risk of Recurrence in Stage II and III Colorectal Cancer Patients: Results from an International Cohort Consortium
Source: Metabolites. 2021 Feb 24;11(3):129. doi: 10.3390/metabo11030129 (PMC7996362; doi:10.3390/metabo11030129)
Supplement: Supplementary file 1 [file metabolites-11-00129-s001.pdf]

Supplementary Table 1. Metabolite classes and biochemical names of metabolites included for the present study.

Metabolite class	Biochemical name	Abbreviation
Acylcarnitines	Carnitine	C0
Acylcarnitines	Tetradecenoylcarnitine	C14:1
Acylcarnitines	Hexadecanoylcarnitine	C16
Acylcarnitines	Hexadecenoylcarnitine	C16:1
Acylcarnitines	Octadecanoylcarnitine	C18
Acylcarnitines	Octadecenoylcarnitine	C18:1
Acylcarnitines	Octadecadienylcarnitine	C18:2
Acylcarnitines	Acetylcarnitine	C2
Acylcarnitines	Propionylcarnitine	C3
Acylcarnitines	Butyrylcarnitine	C4
Acylcarnitines	Valerylcarnitine	C5
Amino acids	Alanine	Ala
Amino acids	Arginine	Arg
Amino acids	Asparagine	Asn
Amino acids	Citrulline	Cit
Amino acids	Glutamine	Gln
Amino acids	Glutamate	Glu
Amino acids	Histidine	His
Amino acids	Isoleucine	Ile
Amino acids	Leucine	Leu
Amino acids	Lysine	Lys
Amino acids	Methionine	Met
Amino acids	Ornithine	Orn
Amino acids	Phenylalanine	Phe
Amino acids	Proline	Pro
Amino acids	Serine	Ser
Amino acids	Threonine	Thr
Amino acids	Tryptophan	Trp
Amino acids	Tyrosine	Tyr
Amino acids	Valine	Val
Biogenic amines	Asymmetric dimethylarginine	ADMA
Biogenic amines	Creatinine	Creatinine
Biogenic amines	Kynurenine	Kynurenine
Biogenic amines	Sarcosine	Sarcosine
Biogenic amines	Symmetric dimethylarginine	SDMA
Biogenic amines	t4-hydroxyproline	t4-OH-Pro
Biogenic amines	Taurine	Taurine
Hexoses	Hexoses	H1
Lysophosphatidylcholines	Lysophosphatidylcholine (acyl) C16:0	lysoPC a C16:0
Lysophosphatidylcholines	Lysophosphatidylcholine (acyl) C16:1	lysoPC a C16:1

Lysophosphatidylcholines	Lysophosphatidylcholine (acyl) C17:0	lysoPC a C17:0
Lysophosphatidylcholines	Lysophosphatidylcholine (acyl) C18:0	lysoPC a C18:0
Lysophosphatidylcholines	Lysophosphatidylcholine (acyl) C18:1	lysoPC a C18:1
Lysophosphatidylcholines	Lysophosphatidylcholine (acyl) C18:2	lysoPC a C18:2
Lysophosphatidylcholines	Lysophosphatidylcholine (acyl) C20:3	lysoPC a C20:3
Lysophosphatidylcholines	Lysophosphatidylcholine (acyl) C20:4	lysoPC a C20:4
Lysophosphatidylcholines	Lysophosphatidylcholine (acyl) C28:1	lysoPC a C28:1
Phospatidylcholines (acyl-alkyl)	Phospatidylcholine (acyl-alkyl) C30:0	PC ae C30:0
Phospatidylcholines (acyl-alkyl)	Phospatidylcholine (acyl-alkyl) C30:2	PC ae C30:2
Phospatidylcholines (acyl-alkyl)	Phospatidylcholine (acyl-alkyl) C32:1	PC ae C32:1
Phospatidylcholines (acyl-alkyl)	Phospatidylcholine (acyl-alkyl) C32:2	PC ae C32:2
Phospatidylcholines (acyl-alkyl)	Phospatidylcholine (acyl-alkyl) C34:0	PC ae C34:0
Phospatidylcholines (acyl-alkyl)	Phospatidylcholine (acyl-alkyl) C34:1	PC ae C34:1
Phospatidylcholines (acyl-alkyl)	Phospatidylcholine (acyl-alkyl) C34:2	PC ae C34:2
Phospatidylcholines (acyl-alkyl)	Phospatidylcholine (acyl-alkyl) C34:3	PC ae C34:3
Phospatidylcholines (acyl-alkyl)	Phospatidylcholine (acyl-alkyl) C36:0	PC ae C36:0
Phospatidylcholines (acyl-alkyl)	Phospatidylcholine (acyl-alkyl) C36:1	PC ae C36:1
Phospatidylcholines (acyl-alkyl)	Phospatidylcholine (acyl-alkyl) C36:2	PC ae C36:2
Phospatidylcholines (acyl-alkyl)	Phospatidylcholine (acyl-alkyl) C36:3	PC ae C36:3
Phospatidylcholines (acyl-alkyl)	Phospatidylcholine (acyl-alkyl) C36:4	PC ae C36:4
Phospatidylcholines (acyl-alkyl)	Phospatidylcholine (acyl-alkyl) C36:5	PC ae C36:5
Phospatidylcholines (acyl-alkyl)	Phospatidylcholine (acyl-alkyl) C38:0	PC ae C38:0
Phospatidylcholines (acyl-alkyl)	Phospatidylcholine (acyl-alkyl) C38:2	PC ae C38:2
Phospatidylcholines (acyl-alkyl)	Phospatidylcholine (acyl-alkyl) C38:3	PC ae C38:3
Phospatidylcholines (acyl-alkyl)	Phospatidylcholine (acyl-alkyl) C38:4	PC ae C38:4
Phospatidylcholines (acyl-alkyl)	Phospatidylcholine (acyl-alkyl) C38:5	PC ae C38:5
Phospatidylcholines (acyl-alkyl)	Phospatidylcholine (acyl-alkyl) C38:6	PC ae C38:6
Phospatidylcholines (acyl-alkyl)	Phospatidylcholine (acyl-alkyl) C40:1	PC ae C40:1
Phospatidylcholines (acyl-alkyl)	Phospatidylcholine (acyl-alkyl) C40:2	PC ae C40:2
Phospatidylcholines (acyl-alkyl)	Phospatidylcholine (acyl-alkyl) C40:3	PC ae C40:3
Phospatidylcholines (acyl-alkyl)	Phospatidylcholine (acyl-alkyl) C40:4	PC ae C40:4
Phospatidylcholines (acyl-alkyl)	Phospatidylcholine (acyl-alkyl) C40:5	PC ae C40:5
Phospatidylcholines (acyl-alkyl)	Phospatidylcholine (acyl-alkyl) C40:6	PC ae C40:6
Phospatidylcholines (acyl-alkyl)	Phospatidylcholine (acyl-alkyl) C42:1	PC ae C42:1
Phospatidylcholines (acyl-alkyl)	Phospatidylcholine (acyl-alkyl) C42:2	PC ae C42:2
Phospatidylcholines (acyl-alkyl)	Phospatidylcholine (acyl-alkyl) C42:3	PC ae C42:3
Phospatidylcholines (acyl-alkyl)	Phospatidylcholine (acyl-alkyl) C42:4	PC ae C42:4
Phospatidylcholines (acyl-alkyl)	Phospatidylcholine (acyl-alkyl) C42:5	PC ae C42:5
Phospatidylcholines (acyl-alkyl)	Phospatidylcholine (acyl-alkyl) C44:3	PC ae C44:3
Phospatidylcholines (acyl-alkyl)	Phospatidylcholine (acyl-alkyl) C44:4	PC ae C44:4
Phospatidylcholines (acyl-alkyl)	Phospatidylcholine (acyl-alkyl) C44:5	PC ae C44:5
Phospatidylcholines (acyl-alkyl)	Phospatidylcholine (acyl-alkyl) C44:6	PC ae C44:6
Phospatidylcholines (diacyl)	Phospatidylcholine (diacyl) C28:1	PC aa C28:1

Phospatidylcholines (diacyl)	Phospatidylcholine (diacyl) C30:0	PC aa C30:0
Phospatidylcholines (diacyl)	Phospatidylcholine (diacyl) C32:0	PC aa C32:0
Phospatidylcholines (diacyl)	Phospatidylcholine (diacyl) C32:1	PC aa C32:1
Phospatidylcholines (diacyl)	Phospatidylcholine (diacyl) C32:2	PC aa C32:2
Phospatidylcholines (diacyl)	Phospatidylcholine (diacyl) C32:3	PC aa C32:3
Phospatidylcholines (diacyl)	Phospatidylcholine (diacyl) C34:1	PC aa C34:1
Phospatidylcholines (diacyl)	Phospatidylcholine (diacyl) C34:2	PC aa C34:2
Phospatidylcholines (diacyl)	Phospatidylcholine (diacyl) C34:3	PC aa C34:3
Phospatidylcholines (diacyl)	Phospatidylcholine (diacyl) C34:4	PC aa C34:4
Phospatidylcholines (diacyl)	Phospatidylcholine (diacyl) C36:0	PC aa C36:0
Phospatidylcholines (diacyl)	Phospatidylcholine (diacyl) C36:1	PC aa C36:1
Phospatidylcholines (diacyl)	Phospatidylcholine (diacyl) C36:2	PC aa C36:2
Phospatidylcholines (diacyl)	Phospatidylcholine (diacyl) C36:3	PC aa C36:3
Phospatidylcholines (diacyl)	Phospatidylcholine (diacyl) C36:4	PC aa C36:4
Phospatidylcholines (diacyl)	Phospatidylcholine (diacyl) C36:5	PC aa C36:5
Phospatidylcholines (diacyl)	Phospatidylcholine (diacyl) C36:6	PC aa C36:6
Phospatidylcholines (diacyl)	Phospatidylcholine (diacyl) C38:0	PC aa C38:0
Phospatidylcholines (diacyl)	Phospatidylcholine (diacyl) C38:3	PC aa C38:3
Phospatidylcholines (diacyl)	Phospatidylcholine (diacyl) C38:4	PC aa C38:4
Phospatidylcholines (diacyl)	Phospatidylcholine (diacyl) C38:5	PC aa C38:5
Phospatidylcholines (diacyl)	Phospatidylcholine (diacyl) C38:6	PC aa C38:6
Phospatidylcholines (diacyl)	Phospatidylcholine (diacyl) C40:2	PC aa C40:2
Phospatidylcholines (diacyl)	Phospatidylcholine (diacyl) C40:3	PC aa C40:3
Phospatidylcholines (diacyl)	Phospatidylcholine (diacyl) C40:4	PC aa C40:4
Phospatidylcholines (diacyl)	Phospatidylcholine (diacyl) C40:5	PC aa C40:5
Phospatidylcholines (diacyl)	Phospatidylcholine (diacyl) C40:6	PC aa C40:6
Phospatidylcholines (diacyl)	Phospatidylcholine (diacyl) C42:0	PC aa C42:0
Phospatidylcholines (diacyl)	Phospatidylcholine (diacyl) C42:1	PC aa C42:1
Phospatidylcholines (diacyl)	Phospatidylcholine (diacyl) C42:2	PC aa C42:2
Phospatidylcholines (diacyl)	Phospatidylcholine (diacyl) C42:5	PC aa C42:5
Phospatidylcholines (diacyl)	Phospatidylcholine (diacyl) C42:6	PC aa C42:6
Sphingolipids	Hydroxysphingomyeline C14:1	SM (OH) C14:1
Sphingolipids	Hydroxysphingomyeline C16:1	SM (OH) C16:1
Sphingolipids	Hydroxysphingomyeline C22:1	SM (OH) C22:1
Sphingolipids	Hydroxysphingomyeline C22:2	SM (OH) C22:2
Sphingolipids	Hydroxysphingomyeline C24:1	SM (OH) C24:1
Sphingolipids	Sphingomyeline C16:0	SM C16:0
Sphingolipids	Sphingomyeline C16:1	SM C16:1
Sphingolipids	Sphingomyeline C18:0	SM C18:0
Sphingolipids	Sphingomyeline C18:1	SM C18:1
Sphingolipids	Sphingomyeline C20:0	SM C20:2
Sphingolipids	Sphingomyeline C24:0	SM C24:0
Sphingolipids	Sphingomyeline C24:1	SM C24:1

Sphingolipids	Sphingomyeline C26:0	SM C26:0
Sphingolipids	Sphingomyeline C26:1	SM C26:1
